# From images to physics-based computational models to digital twins: a framework for personalized cancer therapies

**DOI:** 10.3389/fradi.2026.1737577

**Published:** 2026-02-09

**Authors:** Farshad Moradi Kashkooli, Wenbo Zhan, Ajay Bhandari, Tahir I. Yusufaly, Michael C. Kolios, Arman Rahmim, M. Soltani

**Affiliations:** 1 Department of Physics, Toronto Metropolitan University, Toronto, ON, Canada; 2 School of Engineering, King’s College, University of Aberdeen, Aberdeen, United Kingdom; 3 Biofluids Research Lab, Department of Mechanical Engineering, Indian Institute of Technology (Indian School of Mines), Dhanbad, India; 4 Division of Radiology and Radiological Sciences, Johns Hopkins School of Medicine, Baltimore, MD, United States; 5 Departments of Radiology and Physics, University of British Columbia, Vancouver, BC, Canada; 6 Department of Basic and Translational Research, BC Cancer Research Institute, Vancouver, BC, Canada; 7 Department of Electrical and Computer Engineering, University of Waterloo, Waterloo, ON, Canada; 8 Department of Mechanical Engineering, K.N. Toosi University of Technology, Tehran, Iran

**Keywords:** computational modeling, digital twins, drug delivery, image-based models, medical imaging, multi-scale and multi-physics models, nanomedicine, personalized medicine

## Abstract

In this work, we highlight recent advances in computational modeling that have significantly enhanced prospects of personalized cancer therapies by enabling insightful integration of patient-specific data, including medical images. Computational models, encompassing multi-physics and multi-scale approaches, can simulate drug transport and interactions within tissues and environments, including the tumor microenvironment, and facilitate the development of targeted diagnostic and therapeutic strategies. The incorporation of machine learning algorithms has further refined modeling, improving predictive accuracy and enabling real-time adaptive treatment planning. Although challenges remain in model validation and clinical translation, ongoing advancements are steadily bridging these gaps, bringing computational models and technologies closer to routine clinical application for the improvement of patient outcomes.

## 1 Introduction

Computational models play an increasingly important role in healthcare, particularly in cancer treatment. Through the simulation of complex biological processes, modeling can facilitate more efficient and cost-effective drug development, clinical trials, and personalization of diagnostic and therapeutic delivery (1, 2). Recent guidelines and regulations from the U.S. FDA (3, 4) and the EU REACH initiative (5) highlight how regulatory agencies have increasingly come to accept computational models as substitutes for animal testing (6) during the early assessment of drug safety and efficacy (7, 8). This might enable one day drugs to advance to human trials based entirely on computational modeling evaluations.

The modeling and simulation of biological and physiological environments is a complex task, requiring both multi-physics (integrating multiple physics principles governing the system) and multi-scale (integrating multiple scales from the macro to micro level) approaches (9–11). As an example, consider the problem of simulating therapeutic [e.g., drugs, nanoparticles (12)] or diagnostic [e.g., PET radiotracer (13)] agents’ distribution in a solid tumor. The underlying governing equations originate from diverse areas of expertise, including pharmacokinetics, pharmacodynamics, fluid mechanics, tissue biomechanics, heat and mass transfer, and biochemical processes. At the macroscopic level, one must consider variables such as interstitial fluid flow characteristics and the concentration of the drug across the tumor. Simultaneously, at the microscopic scale, detailed features including microvascular structure and density, blood perfusion in microvessels, interactions at the vascular wall, and drug-cell interactions of need to be accounted for (14).

Recently, there has been a significant push to extend computational cancer modeling approaches in a manner that pairs simulation with continuous collection of patient-specific data, thereby enabling the creation of biomedical “digital twins (DT)” for clinical applications (15–22). A DT is a dynamic virtual model that replicates the structure, context, and behavior of a physical, biological, and/or engineered system while being iteratively updated with real-time data from its physical counterpart (17, 20, 23). This *bidirectional* interaction ensures that data from the physical system refines the virtual model, while predictions and simulations from the DT inform interventions in the real world. In oncology, for example, a DT of breast cancer may integrate serial multimodal imaging (CT, multiparametric MRI, PET) with mechanistic models of tumor growth, vascular permeability, and drug transport to forecast individualized responses to alternative therapies (20, 24). Such patient-specific, data-assimilative updating functionally distinguishes DTs from traditional mathematical models, which operate with fixed parameters and static, single-run simulations. In this way, DTs integrate observations from sensors, imaging, and operational logs to enhance predictive accuracy and decision-making, and can operate autonomously or with human oversight to optimize intervention performance. To ensure reliability towards ultimate deployment and use, it is important to perform verification, validation and uncertainty quantification (VVUQ) (25, 26), as elaborated later. In parallel, recent years have witnessed coordinated international efforts to advance multiscale cancer modeling and *in silico* oncology, including dedicated research collections and initiatives centered on digital and virtual twins for cancer applications (18, 27–30).

A crucial enabler of biomedical DTs is medical imaging and image processing, which provides patient-specific parameters that enhance the accuracy of computational models. Advanced imaging modalities such as MRI, CT, and PET offer critical inputs for constructing personalized DTs, supporting precise drug delivery planning and individualized therapeutic interventions. Beyond raw imaging data, recent studies have explored imaging-derived phenotypes (IDPs) [e.g., (31, 32)] as an additional means of summarizing imaging information for DT personalization in oncology. IDPs refer to quantitative phenotypic descriptors extracted from medical images using dedicated processing pipelines, which carry biological and clinical significance and can be incorporated into biomedical modeling tasks (33, 34). More broadly, quantitative features derived from medical images—including radiomics-based measures, functional imaging biomarkers, and AI-assisted image representations—can capture anatomical and functional characteristics such as tumor morphology, heterogeneity, vascularity, and microenvironmental properties (35–37). By enabling non-invasive inference of biological states and prediction of treatment response, such imaging-informed features can support model calibration and adaptive updating, thereby linking diagnostic imaging with multiscale computational modeling and precision clinical decision-making.

By combining real-time imaging data with computational modeling, DTs hold promise in transforming precision medicine, optimizing drug delivery, and improving overall clinical decision-making (15, 16, 24, 38, 39). In this context, “optimizing drug delivery” refers to improving drug dosing and temporal scheduling, inter-dose intervals, sequencing of multimodal therapies, spatial targeting accuracy, intra-tumoral distribution efficiency, and, when relevant, selecting the most appropriate delivery modality (e.g., intravenous, intra-arterial, intratumoral, or convection-enhanced delivery). These optimization goals are informed by DT-derived predictions of pharmacokinetics, pharmacodynamics, tumor biomechanics, and microenvironmental evolution.

In this work, we argue that image-guided, physics-grounded DTs can operationalize precision oncology by (i) parameterizing image-based drug delivery simulations, (ii) accelerating inference and control with machine learning (ML), and (iii) building clinical trust via VVUQ and translation criteria. We begin in section 2 by defining a unifying framework and scope, and subsequently situate computational modeling within precision oncology in section 3. In section 4, we focus our attention on image-based drug delivery simulations and computational pharmaceutics, specifically, and then describe how ML [including physics-informed neural networks (PINNs)] can be integrated with these models in section 5. In sections 6 and 7, we discuss requirements for VVUQ and clinical translation, respectively. Section 8 concludes the manuscript, offering an outlook and future perspectives.

## 2 A unifying framework and scope

This perspective is anchored around a delivery pipeline deeply informed by computational modeling that we call Image-Guided Digital-Twin based Drug/Diagnostic-agent Delivery (IG-DT-DD). To help organize the diverse advances discussed throughout this perspective study, this IG-DT-DD framework is introduced here as a conceptual roadmap that will be used to structure and interpret the advances in computational modeling for personalized cancer therapy reviewed in the following section and beyond. IG-DT-DD is organized around a core image-guided modeling pipeline (elements i–ii), upon which additional DT capabilities—such as ML-assisted inference, VVUQ, and clinical decision support (elements iii–v)—are layered. Key elements of IG-DT-DD include:

- **Patient-specific imaging towards parameter extraction:** from patient imaging, we extract geometric (e.g., segmentation-derived anatomy), transport (e.g., perfusion, diffusion, permeability), mechanical (e.g., tissue stiffness), and spatial heterogeneity (e.g., voxel-level variations in cellularity or vascular density) parameters that help better define the tumor microenvironment (TME) and organ at risks (OARs) and serve as inputs to multi-physics models.
- **Physics-based multi-scale computational modeling and simulation:** vascular, interstitial, and cellular transport (*via* convection–diffusion–reaction) coupled with other biophysical processes (e.g., bioheat transfer), tissue mechanics, as well as pharmacokinetics/pharmacodynamics (PK/PD) and physiologically based pharmacokinetic (PBPK) models are all integrated in a multi-physics setting to generate predictive spatiotemporal simulations of drug delivery.
- **ML-assisted inference and control:** accelerated and scalable algorithms for parameter estimation, surrogate modeling, and numerical optimization allow for adaptive dosing.
- **VVUQ**: computational models are verified numerically and validated with ground-truth data, from which one can quantify uncertainty and assess confidence in the results.
- **Clinical decision support:** interpretable indices for recommended, more-optimal therapies are provided.

**Figure 1:**
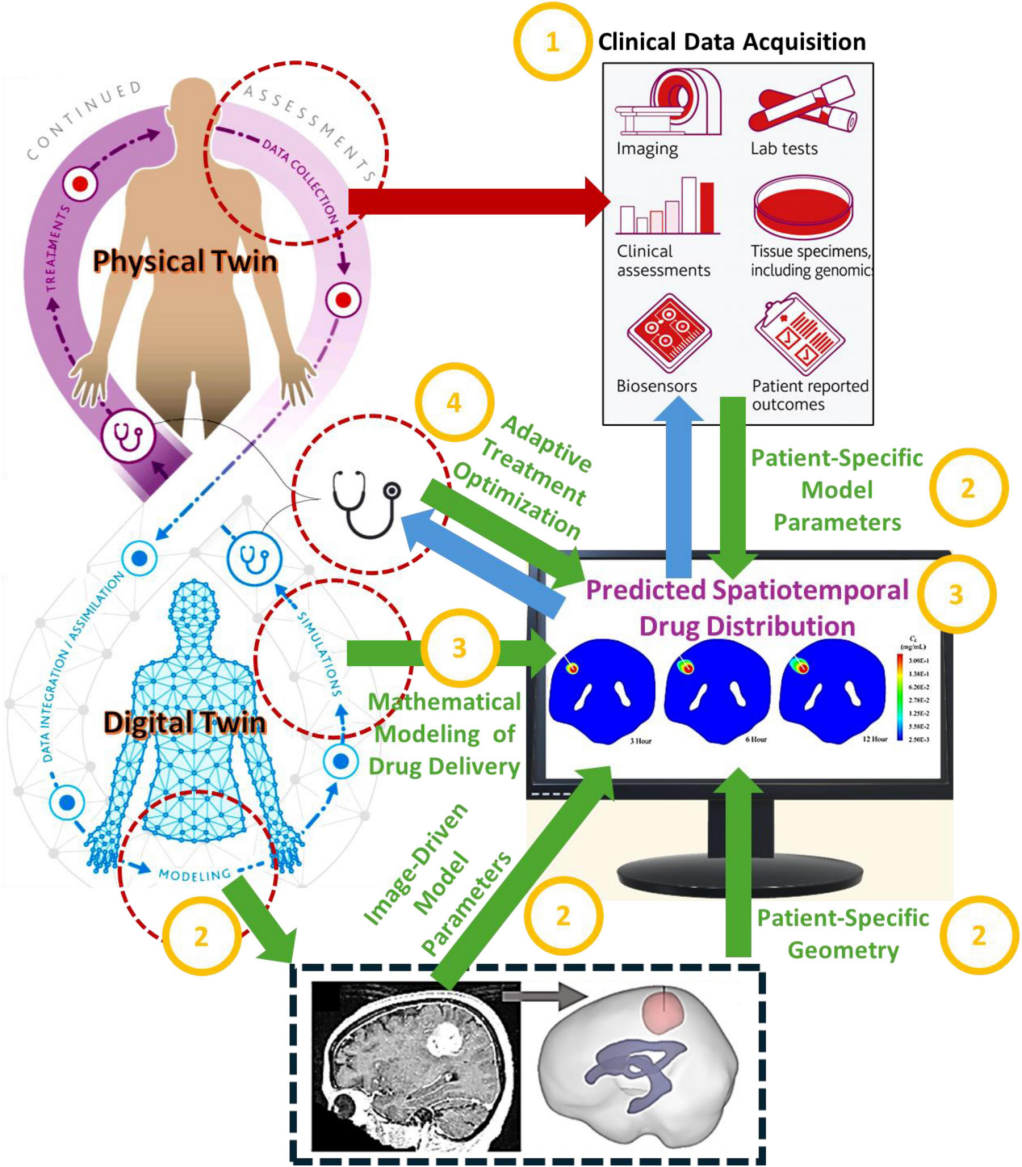
The core image-guided digital twin–based drug delivery (IG-DT-DD) workflow. (1) Clinical data acquisition: Multimodal patient data from the physical twin—including medical imaging, biospecimens, laboratory tests, biosensors, and clinical assessments—provide individualized inputs to the framework. (2) Parallel parameter and geometry extraction [Stage (i) in the text]: Imaging and clinical data are processed concurrently to derive patient-specific anatomical geometry and biophysical model parameters, enabling individualized DT formulation. (3) DT modeling and simulation [Stage (ii) in the text]: Patient-specific geometry and parameters are integrated into mechanistic multiphysics models to generate predictive spatiotemporal drug delivery simulations within the tumor and surrounding tissues. (4) Model-informed treatment planning and adaptive optimization: Simulation outputs support personalized therapeutic decision-making and provide feedback to the physical twin, completing the iterative digital–physical loop. Digital-twin capabilities described as Stages (iii)–(v) in the text—ML-assisted inference and control, VVUQ, and clinical decision support—operate across and build upon Steps (2)–(4) in the figure, rather than constituting additional sequential pipeline steps. Elements of this schematic were adapted, with permission, from previously published sources (23, 40).

This pipeline provides a foundational framework that serves as a foundation for precision oncology by enabling patient-specific personalization via computational modeling. Figure 1 illustrates how multimodal experimental, preclinical, clinical, and patient-derived data are systematically integrated into image-guided DT models to generate predictive simulations of therapy response. This image-guided modeling stage constitutes the core of IG-DT-DD and establishes the basis for subsequent, more advanced DT capabilities, including ML integration, VVUQ, and clinical decision support.

## 3 Advances in computational modeling for personalized cancer therapy

Building on the unifying framework introduced in the previous section, computational modeling promises to be especially beneficial in personalized cancer therapy, also known as precision oncology. Precision oncology focuses on understanding and predicting individual characteristics of patient tissues and tumors, beyond conventional “one-size-fits-all” descriptions of cancer biology, to improve therapeutic outcomes (41). Computational modeling and biomedical DTs offer a non-invasive way to understand and predict cancer development, progression, and therapeutic response, thus aiding medical practitioners in making informed decisions regarding treatment strategies. Meanwhile, complete personalization of cancer therapy via computational models is a challenging task due to extensive heterogeneities at different scales.

Nevertheless, with advancements in medical technology and computing methods, strategies for developing personalized models to guide clinical decision-making have become feasible in principle (11). Advances in medical imaging and image processing have been instrumental in driving image-based computational modeling for precision oncology. A tangentially related example of this is radiation therapy treatment planning, which generates a digital representation of a patient's anatomy from computed tomography (CT) or magnetic resonance imaging (MRI) to compute and optimize dose delivery—an imaging-driven, model-based workflow that mirrors key DT concepts (20). This clinically established workflow already integrates real patient imaging data. In high-grade gliomas, for instance, DT-like modeling relies on serial imaging: pretreatment MRI establishes the tumor's initial anatomical and biological conditions, while follow-up scans enable calibration of patient-specific radiotherapy response (20). Recent work employing weekly quantitative MRI to update mechanistic tumor-response models demonstrates how retrospective clinical datasets can be coupled with in silico simulations to iteratively calibrate patient-specific digital representations of therapy (42). Although such frequent imaging is not yet standard practice, emerging MRI-guided linear accelerator platforms (43) enable routine, high-frequency anatomical and functional imaging, strengthening the feasibility of clinically deployable glioma DTs.

Building on these DT-aligned, imaging-driven principles, the development of personalized computational cancer models and virtual clinical trials necessitates the incorporation of powerful and quantitative imaging modalities (44), such as MRI, CT, and positron emission tomography (PET). These modalities, frequently using specialized imaging sequences (44), can yield information on patient-specific features of the TME that, when coupled with continuum models, help predict patient-specific and optimized drug delivery outcomes (45), drug deposition and washout patterns (46), combined treatment strategies (47), and the prognosis of tumor growth and metastasis (48). Such imaging of the TME can also be paired with complementary computational modeling approaches, such as OncoKB (49), that focus on incorporating genomic data and bioinformatics to predict mutations and significant molecular signatures (biomarkers) that modulate drug delivery, tumor response, and therapeutic outcomes (50). Very recently, these converging advances have enabled the creation of virtual tumor models—tumor DTs—that integrate medical imaging, clinical history, genomic profiles, and ML for an individual patient (51, 52). This helps optimize treatment strategies on virtual models before administering the treatment to a specific patient.

Computational modeling approaches are thus gaining traction and have transformative potential for precision oncology. However, clinical translation remains challenging due to several factors, including model validation, ethical considerations, complex tumor biology, and extreme variability in integrated data. Robust clinical deployment requires multi-stage verification and validation—spanning internal and external retrospective testing, prospective evaluation, and explicit quantification of model uncertainty and parameter identifiability—to ensure that predictions remain reliable across patient populations (53, 54). Ethical considerations further include responsible use and governance of patient data, safeguards against algorithmic bias that may impact underrepresented groups, and maintaining interpretability and human-in-the-loop oversight so that model-derived recommendations remain clinically accountable (55–57). Nonetheless, with enhancements in computing power and big data analytics, computational models are expected to provide cancer patients with precise and personalized treatments in the future.

Having established why imaging-personalized models matter in precision oncology, we now explore image-based drug delivery simulations and their role in computational pharmaceutics/oncology.

## 4 Image-based drug delivery simulations and computational pharmaceutics

Image-based simulations correspond to early stages of the IG-DT-DD pipeline—specifically, (1) patient-specific imaging and parameter extraction and (2) physics-based multi-scale computational modeling and simulation. These stages jointly enable quantitative, image-driven representations of anatomy, biophysical parameters, and transport dynamics, forming the foundation on which delivery simulations are built. By bridging laboratory studies and clinical trials, image-based simulations provide a controlled and cost-effective environment to explore delivery strategies, identify failure modes, and reveal determinants of transport inefficiency. Most current efforts in this area remain *in silico* or preclinical, though several groups now integrate trial- or patient-imaging datasets to enhance clinical realism and personalize model predictions (44, 58, 59). When coupled with patient or trial data, these models support rigorous optimization of drug delivery. Clinically, ongoing developments could enable patient-specific treatment planning and real-time adaptive control of therapy, advancing both safety and efficacy. Recent papers have emphasized the importance of coupling image-derived parameters with mechanistic transport models to overcome biological delivery barriers and improve therapeutic efficacy (18, 44, 60).

The development of personalized computational models from medical imaging data typically requires a sequence of steps, including anatomical segmentation, interpolation of missing or intermediate slices, surface reconstruction, volumetric mesh generation, and mapping of patient-specific data onto the reconstructed three-dimensional domain. Although a wide range of classical and AI–based tools exist to address individual stages of this pipeline, they are commonly integrated through complex, multi-software workflows. To overcome these limitations, for example, the authors of (61) proposed an end-to-end framework implemented as a Python library, im2mesh, which automates the entire process from clinical image acquisition to the generation of patient-specific 3D finite element meshes with interpolated imaging-derived data.

Computational pharmaceutics represents a new paradigm in drug delivery, bridging the gap between pharmaceutics and molecular modeling at various scales. This approach covers aspects such as nanoparticle-based drug delivery (18), computational prediction of drug solubility, and the ability to cross physiological drug delivery obstacles, including the blood-brain barrier, among others. These advancements contribute to the development of more effective and targeted drug delivery systems. However, achieving high resolution and predictive accuracy while maintaining computational efficiency remains challenging for these models. Addressing these demands requires a robust modeling framework that balances precision and speed. Accurate parameterization of TME and OARs characteristics, diagnostic agents (e.g., PET radiotracers, microbubbles/nanobubbles), therapeutic agents (e.g., drugs, nanoparticles, radiopharmaceuticals), and external (e.g., ultrasound or magnetic field for drug release) and internal (e.g., pH for drug release) sources is essential but complex, making validation challenging. Verifying model assumptions and simplifications is also necessary to support effective diagnostic and therapeutic decisions.

Because high-fidelity solvers—i.e., models that resolve fine spatial and temporal scales and capture detailed multi-physics or multi-scale biological processes— are often too slow and computationally intensive for clinic-timescale applications, we discuss ML integration to demonstrate how learning augments and accelerates physics models without sacrificing mechanistic interpretability.

## 5 Integration of machine learning

Artificial intelligence (AI)-driven, data-based approaches such as ML have inaugurated a new paradigm in oncology, enhancing mechanistic modeling and enabling major advances IN cancer diagnosis, therapy, and drug discovery (62). ML is a branch of AI that employs computational algorithms to identify patterns in datasets without minimizing the need for explicitly programmed instructions or task-specific rules (63). The integration of ML algorithms with multi-scale and multi-physics models holds promise for bridging existing biomedical knowledge gaps and thereby enhancing the therapeutic precision of cancer treatment. As a purely data-driven approach to prediction and informed decision-making, ML is noted for its flexibility and adaptability compared to model-based approaches. For example, an important clinical challenge in drug delivery is the ability to adjust strategies in real-time based on patient conditions. Traditional numerical methods depend on predefined initial parameters and their variations, which cannot be modified during simulation. In contrast, ML, with its high adaptability, enables dynamic adjustments to drug delivery strategies (64–66) by incorporating real-time monitoring data. This ensures optimal therapeutic outcomes while minimizing adverse effects. Such a feedback-driven and adaptive approach holds significant potential for advancing personalized dosing strategies in clinical settings.

ML's ability to handle extensive datasets also makes it a valuable tool for analysing high-throughput experimental datasets, including pharmaceutical (67–69) and imaging data (70). For medical imaging, especially, ML algorithms are increasingly utilized to efficiently process large volumes of medical imaging data, assisting clinicians in making accurate and rapid diagnostic decisions (71, 72). These non-invasive imaging techniques provide valuable insights into patients’ state, encompassing anatomical structures, physiological functions, and pathological alterations (44). This includes detailed visualization of organs (*e.g.*, brain, heart, lungs), vascular networks and blood flow dynamics, metabolic activity in tissues (e.g., PET-based tumor profiling and absorbed dose), and microstructural changes such as fibrosis or edema. By developing ML algorithms based on established image-processing models (22), quantitative parameters such as such as perfusion, permeability, apparent diffusion coefficients, vascular density estimates, and surrogate measures of tissue stiffness can be extracted from imaging data sets (18, 44). These parameters provide actionable biomarkers for cancer evaluation, prognosis, and treatment-response assessment. Radiomics-based ML approaches, for example, have demonstrated the ability to extract high-dimensional quantitative features from CT—including intensity, shape, and texture—that carry independent prognostic value across cancer types (37). Likewise, deep-learning models have achieved automated segmentation of contrast-enhancing and non-enhancing tumor components on MRI, enabling quantitative assessment of longitudinal tumour-volume dynamics and providing response evaluations that outperform manual Response Assessment in Neuro-Oncology (RANO) criteria (73). These parameters can subsequently serve as inputs for predictive models, thereby enhancing their accuracy and robustness (74–76). Moreover, integrating individualized patient data further facilitates the personalization of these ML models.

Current image-based modeling often relies on numerical methods, such as the finite volume method (77) and finite element method (77), to solve complex equations (2, 78). These methods are computationally intensive, often requiring hours or days to complete, which is impractical for clinical applications. ML's computational efficiency offers faster predictions. However, the complexity of drug delivery processes, coupled with limited training data derived from imaging parameters and experimental results, poses challenges for traditional ML models. Data scarcity, especially for *in vivo* drug delivery outcomes, further limits their applicability. To address these gaps, physics-informed neural networks (PINNs) have emerged as a promising approach (79). By developing algorithms based on fundamental principles from physics, chemistry, and physiology into ML algorithms, PINNs leverage established mathematical models to enhance predictive accuracy, even with limited data. Recent work has demonstrated how physics-informed DT frameworks can integrate longitudinal patient data to infer tumor evolution and guide personalized therapy, highlighting the clinical potential of ML-enhanced DTs (60). Relatedly, the burgeoning areas of scientific machine learning (SciML) and differentiable programming, along with strategies such as active learning, offer promise for seamlessly integrating deep learning architectures with parameter inference schemes, effectively combining the rigor of mechanistic modeling with the flexibility of modern AI to deliver the best of both worlds (80).

Speed alone does not confer clinical credibility; what matters is how models are rigorously verified, validated, and characterized by their uncertainties: principles that lie at the heart of VVUQ.

## 6 Considerations for model verification, validation, and uncertainty quantification (VVUQ)

Mathematical models and simulations must undergo empirical validation before being accepted for clinical application. Within the 3-tier framework of VVUQ, (i) verification ensures mathematical and computational correctness, (ii) validation tests predictive fidelity against experimental or clinical data, and (iii) uncertainty quantification evaluates the reliability of outputs. The complex physiological and physicochemical processes involved in drug delivery (14) often make it challenging to describe these processes using a single theoretical formula. As a result, image-based drug delivery simulations require numerous model parameters. Some of these parameters can be extracted from medical imaging data [e.g., notably for radiopharmaceutical therapy (RPT) agents with the theranostics property of being also imaged with PET or SPECT imaging (81)], while others should be sourced from additional data, including representative parameter values derived from experiments using different species of animal, literature, or reasonable assumptions based on existing knowledge (25, 47, 82, 83). Consequently, simulation results typically align only qualitatively with experimental and clinical observations of the spatiotemporal drug distribution. In such cases, the model together with its parameters can only be used for comparative analysis between different results. These comparisons are valuable for determining the influence of specific variables on drug delivery outcomes and identifying potential opportunities to improve delivery strategies.

Currently, the experimental data used for model validation often involve fluorescent agents (84), or imaging contrasts (47). Compared to drugs used in clinical practice, these agents may involve simpler physicochemical processes, particularly with fewer biochemical reactions. These surrogates simplify the underlying physicochemical and biochemical processes, highlighting a limitation of the validation stage in VVUQ: benchmark data may not adequately reflect the drugs used clinically. As such, the credibility of model validation using these experimental results leaves room for improvement. Additionally, current simulations commonly focus on the mean concentration of the studied drugs (14), without considering spatiotemporal variations that are still difficult to observe in clinical or animal experiments. Such gaps underscore the need for broader VVUQ practices, including experimental designs that capture spatiotemporal dynamics and more representative validation datasets. In RPTs, such assessments can be performed more easily via imaging radiopharmaceuticals as theranostic counterparts to therapy radiopharmaceuticals (85, 86).

Generalizing across tumor treatment models, VVUQ can be summarized in three core stages (26, 54): (i) verification of structural fidelity and numerical implementation (i.e., “to build the model right”), (ii) validation against preclinical and clinical data—both internally (same dataset) and externally (independent dataset)—using fit metrics, model selection, and sensitivity analysis (i.e., “to build the right model”), and (iii) uncertainty quantification to characterize the precision and reliability of predictions (i.e., “to know how right the model is,” for example when elevated interstitial fluid pressure or variable tumor stiffness alter therapy response). Embedding these three steps enables tumor treatment simulations to move beyond qualitative insights toward clinically credible platforms that deliver accuracy, robustness, and interpretable indices—ultimately supporting precision oncology and patient-specific decision making.

## 7 Clinical translation requirements

Clinical translation is one of the key directions for the development of image-based drug delivery modeling. Achieving this goal requires models to meet several essential criteria:

*Accuracy*: The model must provide sufficiently accurate predictions. This necessitates thorough validation. However, it is important to note that the required level of accuracy may vary depending on the purpose. For example, qualitative simulation results can be used to assess the impact of different parameters and design patient-specific treatment plans based on existing delivery protocols. In contrast, real-time adjustments to drug delivery strategies in clinical practice require more precise quantitative predictions.
*Efficiency:* The model should be sufficiently fast and computationally inexpensive to ensure that predictions can be generated within an acceptable timeframe for clinical intervention. Achieving this requires simplifying the model while maintaining accuracy. To this end, model reduction methods (87) based on sensitivity analysis and Fisher Information Matrix spectral methods (88) offer promising strategies. Integrating AI may also provide an effective solution.
*Robustness*: The model needs to be stable enough to handle the complexities of clinical scenarios. It should comprehensively account for the key aspects of drug delivery processes and the primary influencing factors for specific diseases or drugs. Furthermore, as an open system, the model should allow for continuous updates and improvements to enhance its functionality.
*Clinically Relevant Indices*: The model must provide measurable indices of drug delivery outcomes. These indices should intuitively describe the spatial and temporal distribution of drug effects, enabling clinicians to make informed decisions.

We add that the field of *implementation science* (IS) has a significant role to play in this very space (89). Most advanced computational tools fail to gain traction in clinical practice at all or in a timely fashion. More broadly, evidence has shown an average 17-year lag between generating scientific knowledge and implementing it in routine care (90). IS offers a practical, evidence-based approach to close this gap, using systematic frameworks, targeted strategies, and hybrid research designs to accelerate the integration of computational models into clinical imaging workflows. Notably, IS recognizes that sufficient “reason to use” and evidence for value of a powerful solution is only the first step in translation, and one has to also address “means to use” (e.g., infrastructure challenges), “methods to use” (workflow compatibility issues), and “desire to use” (lack of trust, fears of workflow disruption, and medicolegal implications) (91).

A powerful solution within IS is integrated knowledge translation (iKT) (92), which stresses the importance of engaging knowledge users—clinicians, administrators, patients, and decision-makers—early and continuously throughout the research process. Instead of treating end users as passive recipients of innovation, iKT positions them as active co-designers and co-implementers from the outset. This collaborative approach enhances relevance, fosters co-creation, and improves readiness for adoption. Ultimately, applying IS is not merely a methodological advance; it is a need to accelerate translation of computational models and improve patient outcomes.

## 8 Looking beyond the horizon & future perspectives

Computational modeling is expected to become a cornerstone of clinical oncology, particularly through the advancement of DTs—virtual representations of individual patients that continuously update with multimodal data. By integrating imaging, genomic, and clinical information, such models can simulate tumor behavior, predict treatment response, and guide personalized planning of treatment. This “*in silico* rehearsal” of therapeutic strategies potentially offers to reduce clinical trial-and-error, accelerate decision-making, and improve patient outcomes.

Within the IG-DT-DD pipeline, DTs unify four critical domains: image-derived parameters, physics-based multi-scale modeling, ML–driven acceleration, and VVUQ for clinical readiness. Together, these elements enable simulations that are both biologically faithful and computationally feasible. The next frontier lies in translating these advances into clinical workflows, ensuring that model predictions are not only accurate but also clinically relevant.

ML will continue to refine predictive accuracy, particularly through emerging approaches such as PINNs and SciML. These methods offer scalable solutions for real-time therapy adaptation while preserving mechanistic insight. However, persistent challenges remain, including limited *in vivo* datasets for training and validation, the complexity of tumor heterogeneity across scales, and the need for robust, transparent uncertainty quantification. Addressing these challenges requires close collaboration among computational scientists, clinicians, and regulatory agencies to ensure that models are trustworthy and ready for clinical use.

Ultimately, the IG-DT-DD pipeline provides a cohesive roadmap for advancing precision oncology. By embedding imaging, physics, and ML within a validated DT infrastructure, it becomes possible to design patient-specific treatment strategies that are continuously optimized over the course of therapy. With sustained progress in imaging technology, scalable computation, and rigorous validation, computational models are poised to transform cancer care from generalized protocols to truly personalized medicine.

## Data Availability

The original contributions presented in the study are included in the article/Supplementary Material, further inquiries can be directed to the corresponding author.
